# Effect of Structured Perioperative Education on Postoperative Pain Following Lung Cancer Surgery: A Prospective Non-randomized Interventional Study With 12-Month Follow-Up

**DOI:** 10.7759/cureus.110462

**Published:** 2026-06-08

**Authors:** Anastasios N Moysiadis, Maria Kalafati, Alexandra - Stavroula G Nieri, Konstantinos Kontzoglou, Dimitrios Dimitroulis, Sofoklis Mitsos, Ioannis Tomos, Nikolaos Korodimos, Nikolaos Chondropoulos, Periklis Tomos

**Affiliations:** 1 Department of Thoracic Surgery, Attikon University Hospital, National and Kapodistrian University of Athens, Athens, GRC; 2 Department of Nursing, National and Kapodistrian University of Athens, Athens, GRC; 3 Second Department of Propaedeutic Surgery, National and Kapodistrian University of Athens, Athens, GRC; 4 Department of Pulmonology, Sotiria Hospital, Athens, GRC; 5 Department of Thoracic Surgery, Eugenideio Therapeutirio 'Agia Trias' Hospital, Athens, GRC

**Keywords:** lung cancer, pain management, patient education, perioperative education, postoperative pain, rehabilitation, supportive care, thoracic oncology, thoracic surgery

## Abstract

Introduction: Postoperative pain remains a major challenge after lung cancer surgery and may negatively affect recovery, functional status, and quality of life. Structured perioperative educational interventions may improve symptom management and postoperative outcomes. This study evaluated the effect of a structured educational intervention on postoperative pain outcomes in patients undergoing lung cancer surgery.

Methods: This prospective, single-center, non-randomized interventional study included 160 patients undergoing surgical treatment for lung cancer between October 2021 and March 2024. Participants were allocated to either an intervention group (n=80), which received structured perioperative education in addition to usual care, or a control group (n=80), which received usual care alone. The educational intervention was delivered preoperatively and reinforced during the first three postoperative days, with caregiver participation encouraged. Pain outcomes were assessed preoperatively and at 1, 6, and 12 months postoperatively using the Greek-language Pain Rating Scale. The primary outcome was current pain intensity. Secondary outcomes included average pain intensity during the previous week, pain distress, and pain-related interference with daily activities. Longitudinal analyses were performed using linear mixed-effects models.

Results: Significant group, time, and group × time interaction effects were observed for all pain-related outcomes (all p<0.001). Compared with the control group, patients receiving the educational intervention demonstrated significantly lower postoperative pain intensity, reduced pain distress, and less pain-related interference with daily activities across follow-up assessments. The largest between-group differences were generally observed at six months postoperatively. Sensitivity analyses adjusting for baseline pain scores, length of hospital stay, and immunotherapy confirmed the robustness of the findings.

Conclusion: Structured perioperative education was associated with improved postoperative pain outcomes following lung cancer surgery. Educational interventions incorporating caregiver involvement and reinforcement during the early postoperative period may represent a feasible and low-risk supportive strategy to improve postoperative recovery and pain management in patients undergoing thoracic surgery. Further randomized multicenter studies are warranted to confirm these findings.

## Introduction

Lung cancer remains a major global health challenge and continues to be one of the leading causes of cancer-related mortality worldwide [1]. In addition to its high mortality, the disease imposes a substantial physical, psychological, and social burden on patients, significantly affecting overall quality of life. Symptoms related to the disease itself, combined with treatment-associated complications, often compromise daily functioning and long-term recovery [2].

Several factors influence postoperative recovery and quality of life in patients undergoing thoracic surgery for lung cancer. Recovery outcomes are shaped not only by the type of surgical intervention but also by individual patient characteristics, including age, baseline physical condition, comorbidities, nutritional status, and mental health [2,3]. These factors collectively determine the patient’s rehabilitation trajectory and ability to regain functional independence.

Effective postoperative pain management is a critical component of thoracic surgical care, as uncontrolled pain may directly impair mobility, respiratory function, and psychological well-being. Inadequately controlled pain may limit physical activity, impair breathing exercises, and negatively influence psychological adjustment during recovery. Regular pain assessment through validated tools, such as the Visual Analogue Scale (VAS) and Numerical Rating Scale (NRS), and quality-of-life questionnaires, including the European Organisation for Research and Treatment of Cancer Quality of Life Questionnaire Core 30 (EORTC QLQ-C30) and the lung cancer-specific module (EORTC QLQ-LC13), are designed to evaluate patient-reported outcomes for individuals diagnosed with lung neoplasms [4,5].

Increasing attention has been directed toward supportive care interventions, particularly structured patient education, as an essential strategy in comprehensive lung cancer management [2,4,6-9]. Educational interventions are designed to improve patients’ understanding of their disease, strengthen self-management abilities, and enhance adherence to treatment plans. Patients who are better informed about the recovery process, expected symptoms, possible complications, and self-care strategies are more likely to actively participate in their rehabilitation and make informed decisions that support recovery [2,10].

Preoperative and postoperative education may also significantly influence patients’ emotional responses and pain perception. Evidence suggests that participation in structured educational programs is associated with lower reported pain scores, reduced analgesic requirements, and improved functional status [4,11]. Effective patient education should address anticipated pain progression, respiratory care techniques, prevention of complications such as pulmonary infections, and practical self-management strategies, including relaxation methods, mobility exercises, and proper body positioning.

Patient education can generally be categorized into three main domains. Cognitive education focuses on improving understanding of postoperative pain and adherence to pain management protocols. Behavioral education emphasizes practical interventions such as progressive exercise, breathing techniques, and physical rehabilitation strategies. Emotional support addresses psychological distress, anxiety, and pain-related stress through interventions such as mindfulness, psychotherapy, and relaxation training [2,12]. These multidimensional approaches have been shown to enhance illness acceptance, reduce symptom burden, and improve functional quality-of-life measures.

In addition to education, structured rehabilitation programs that incorporate physical exercise and psychosocial support have demonstrated positive effects on postoperative recovery and overall well-being [12,13]. Improved disease knowledge and supportive care may contribute to reduced anxiety, better treatment compliance, and enhanced quality of life [14-17].

Although advances in surgical, chemotherapeutic, and immunotherapeutic approaches have improved survival rates for patients with lung cancer [18,19], treatment-related burden remains substantial. Consequently, modern lung cancer care increasingly emphasizes not only disease treatment but also interventions aimed at improving symptom control, rehabilitation, and long-term quality of life. Despite increasing evidence supporting patient education and supportive care, their full impact on quality-of-life outcomes remains variably reported, highlighting the need for continued research in this field. Furthermore, evidence regarding the long-term effect of structured perioperative educational interventions on postoperative pain trajectories after lung cancer surgery remains limited.

This study aimed to evaluate the effect of a structured educational intervention on postoperative pain intensity among patients undergoing surgery for lung cancer. Specifically, the study examined changes in pain intensity at 1, 6, and 12 months after surgery and compared pain outcomes between patients who received the educational intervention in addition to usual care and those who received usual care alone.

## Materials and methods

Study design

This single-center, prospective, two-arm, parallel-group, non-randomized interventional study was conducted at the Thoracic Surgery Department of a university hospital. Due to the nature of the educational intervention, blinding of participants and the researcher delivering the intervention was not feasible. However, treating physicians and nursing staff were not informed of participants’ group allocation. Therefore, single blinding was implemented at the level of care providers. The study was reported in accordance with the TREND statement. The TREND statement stands for Transparent Reporting of Evaluations with Nonrandomized Designs. It is an official, 22-item reporting guideline specifically created to improve the completeness and transparency of peer-reviewed publications evaluating behavioral and public health interventions that do not use randomized controlled trials (RCTs) [20].

Study Population and Eligibility Criteria

Patients with lung cancer who were scheduled to undergo surgical treatment were recruited during their preoperative hospitalization between October 2021 and March 2024. Patients were eligible for inclusion if they were older than 18 years, had a diagnosis of lung cancer requiring surgical treatment, provided written informed consent, were able to speak, understand, and read Greek, had a preserved level of consciousness allowing cooperation, and were able to cooperate with the researcher and the healthcare team. Patients were excluded if they did not meet all inclusion criteria, had a chronic condition affecting behavior, lacked a supportive psychosocial network, or were unable to cooperate with the medical and nursing staff. Before enrollment, all eligible patients received written information about the study and provided written informed consent. Consent included permission for the collection of study-related data through personal interviews and review of medical records.

Allocation procedure

Participants were assigned to the intervention or control group using a predefined alternating allocation sequence according to the order of surgery. On each surgical day, eligible patients were allocated alternately to the intervention and control groups. On the following surgical day, the sequence started with the opposite group, and this allocation pattern continued throughout the enrollment period.

Although randomization and allocation concealment were not performed, the predefined alternating allocation sequence provided a structured and transparent method of group assignment and helped maintain balanced group sizes. Reversing the starting group on each subsequent surgical day was intended to minimize systematic allocation patterns related to the order of surgery or recruitment day. However, the possibility of residual selection bias and baseline differences between groups cannot be completely excluded. Therefore, the study should be interpreted as a controlled non-randomized interventional study and was reported in accordance with the TREND statement for non-randomized evaluations of behavioral and public health interventions.

Structured educational intervention

Patients allocated to the intervention group received usual clinical care in addition to a structured educational intervention supported by printed educational material. The intervention was delivered during the preoperative hospitalization period and was reinforced during the first three postoperative days. Caregiver participation in the educational sessions alongside the patient was encouraged whenever feasible in order to support understanding, adherence to recommendations, and continuity of care after discharge.

The educational intervention was individualized and covered key aspects of lung cancer, surgical treatment, and postoperative recovery. Patients received information about lung cancer, disease staging, the planned surgical procedure, postoperative drains, surgical incisions, wound care, mobilization, respiratory exercises, and the expected course of postoperative recovery. Education was also provided on lifestyle-related issues, including nutrition, smoking cessation, alcohol use, physical activity, exercise after surgery, permitted weight limits, and follow-up care.

A dedicated component of the intervention focused specifically on postoperative pain. Patients were educated about pain recognition, timely pain reporting, pain management strategies, relaxation techniques, the importance of adequate sleep, and self-care during recovery. The aim of this component was to improve patients’ understanding of postoperative pain, encourage appropriate communication with healthcare professionals, and promote active participation in pain management during the recovery period.

Throughout the educational sessions, patients and caregivers were encouraged to ask questions, express concerns, and discuss issues related to the disease, surgery, postoperative recovery, pain, and follow-up care. The printed educational material was provided to support recall of the information discussed during the sessions and to serve as a reference for patients and caregivers after discharge.

Control group

Patients allocated to the control group received standard perioperative care according to the routine protocol of the Thoracic Surgery Department. They were assessed at the same predefined time points as the intervention group but did not receive any additional structured education, printed educational material, or individualized educational sessions. This allowed comparison between standard care alone and standard care combined with the educational intervention.

Instruments

Demographic and Clinical Characteristics

Baseline demographic and clinical data were collected using a structured questionnaire administered through personal interviews and review of medical records. The questionnaire included information on age, sex, educational level, marital status, occupational status, hospital admission characteristics, lifestyle-related factors, smoking status, alcohol consumption, physical activity, dietary habits, biometric measurements, and personal and family medical history.

Clinical data related to lung cancer and surgical treatment were also recorded, including diagnosis, type of surgical procedure, postoperative clinical course, and relevant perioperative information. These variables were collected to describe the study population and to examine potential differences between the intervention and control groups.

Pain Assessment

The Greek-language version of the Pain Rating Scale, provided by the British Pain Society “Pain Scales in Multiple Languages” resource, was used to assess pain intensity [21,22]. The assessment included current pain intensity, average pain intensity during the previous week, current pain distress, average pain distress during the previous week, and pain-related interference with daily activities. Pain intensity, pain distress, and pain-related interference were rated on numerical scales ranging from 0 to 10, with higher scores indicating greater pain intensity, greater distress, or greater interference with daily activities, respectively. Pain assessments were performed preoperatively and at 1, 6, and 12 months postoperatively. The main outcomes were differences in pain-related scores between the intervention and control groups at each follow-up point and changes in pain scores over time. The pain assessment scale used in this study is provided in the Appendices.

Data collection

Data were collected at baseline before surgery and at 1, 6, and 12 months after lung cancer surgery. Baseline demographic, clinical, and pain-related data were obtained during preoperative hospitalization through face-to-face interviews and review of medical records. Follow-up assessments were performed at 1, 6, and 12 months, with data collected during scheduled follow-up evaluations or, when an in-person assessment was not feasible, by telephone.

Ethics

The study was registered with the Clinical Trials Registry (Unique Protocol ID: 678/21-12-2020; NCT07614204). The study was approved by the Ethics and Scientific Council of the hospital where the research was conducted (approval number: 678; approval date: 21/12/2020). The study was conducted in accordance with the ethical principles of the Declaration of Helsinki and its later amendments [23]. All eligible patients received written information about the study before enrollment, and written informed consent was obtained prior to data collection. Participation was voluntary, and patients were informed of their right to withdraw from the study at any time without any effect on their medical care. All study-related data were treated as confidential and used exclusively for research purposes. Data collection, storage, and processing were performed in accordance with the General Data Protection Regulation (GDPR) and applicable national legislation on personal data protection.

Statistical analysis

Continuous variables were summarized as mean and standard deviation (SD) or median and interquartile range (IQR), depending on distribution assessed by visual inspection and normality tests. Categorical variables were presented as frequencies and percentages. Baseline characteristics were compared between the intervention and control groups using independent-samples t-tests or Mann-Whitney U tests for continuous variables and chi-square tests or Fisher’s exact tests for categorical variables, as appropriate. A formal a priori sample size calculation was not performed due to the exploratory nature of the study.

The primary outcome was current pain intensity over time, assessed at four time points (preoperatively and at 1, 6, and 12 months postoperatively) as repeated measurements. Secondary outcomes included average pain intensity during the previous week, current pain distress, average pain distress during the previous week, and pain-related interference with daily activities.

Longitudinal changes in pain outcomes were analyzed using linear mixed-effects models to account for within-subject correlations due to repeated measurements. For each outcome, separate models were fitted, including fixed effects for group (intervention versus control), time (categorical: T0, T1, T2, and T3), and the group × time interaction. Participant identification number was included as a random intercept to account for inter-individual variability.

The primary effect of interest was the group × time interaction, which assessed whether trajectories of pain outcomes over time differed between groups. Estimated marginal means were calculated for each group at each time point. When appropriate, pairwise comparisons between groups at each time point were performed with Bonferroni correction to adjust for multiple comparisons. Results are presented as estimated marginal means ± standard errors (SE), mean differences, 95% confidence intervals (CI), and p-values.

Because baseline imbalances were observed for current pain intensity and pain distress, sensitivity analyses were performed using baseline-adjusted linear mixed-effects models. These models excluded baseline outcome values from the dependent variable and included baseline measurements as covariates, alongside group, time, and group × time interaction terms.

Additional sensitivity analyses were performed, adjusting for clinically relevant variables that differed between the intervention and control groups, namely, length of hospital stay and immunotherapy, to assess the robustness of the primary findings.

All statistical tests were two-sided, and a p-value < 0.05 was considered statistically significant. Analyses were performed using IBM SPSS Statistics for Windows version 31.0 (IBM Corp., Armonk, NY).

## Results

**Figure 1:**
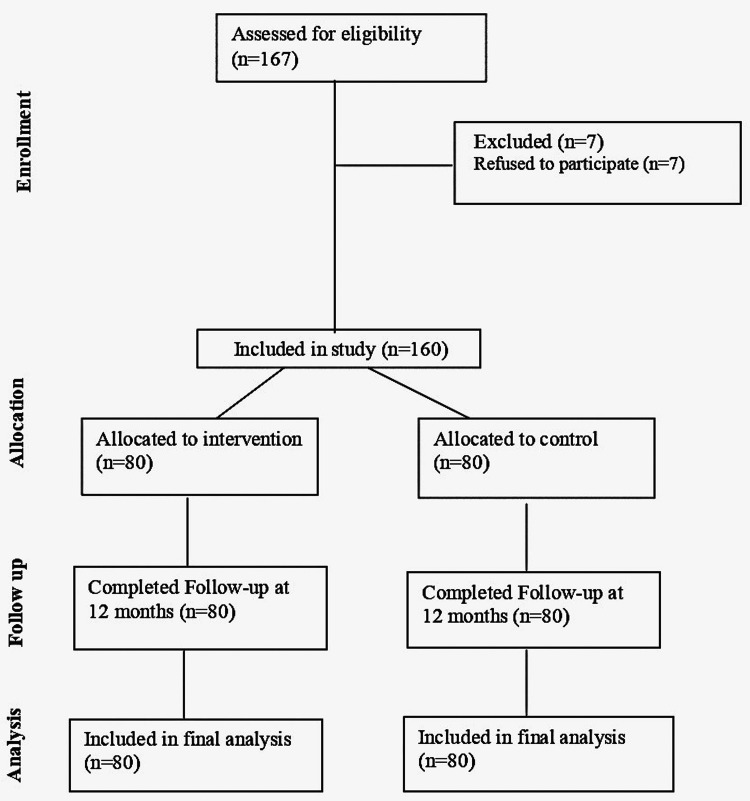
Flow diagram of study participants showing eligibility assessment, exclusion, group allocation, follow-up, and final analysis in a prospective non-randomized interventional study

Overall, 167 eligible patients were approached for participation. Seven patients declined to participate, including four in the control group and three in the intervention group. The final study sample consisted of 160 patients, with 80 patients allocated to the control group and 80 patients allocated to the intervention group. A participant flow diagram is presented in Figure 1.

Demographic and sociodemographic characteristics

The overall sample was predominantly men (104, 65.0%), Greek (110, 68.8%), married (110, 68.8%), and employed (82, 51.2%). The mean age of participants was 66.56±8.39 years.

Baseline sociodemographic characteristics were well balanced between groups, with no statistically significant differences observed in sex (p=0.185), age (p=0.511), nationality (p=0.271), marital status (p=0.682), number of children (p=0.331), educational level (p=0.556), or employment status (p=0.414). These findings suggest that the two groups were broadly comparable at baseline (Table 1).

**Table 1:** Demographic and sociodemographic characteristics of the total sample, control group, and intervention group SD: standard deviation *Monte Carlo

Demographic and sociodemographic characteristics	Total sample	Control group	Intervention group	Comparison, p-value
Sex, number (%)				Χ^2^(1)=1.758; p=0.185
Male	104 (65.0%)	48 (60.0%)	56 (70.0%)	
Female	56 (35.0%)	32 (40.0%)	24 (30.0%)	
Age (years), mean (±SD)	66.56 (±8.39)	66.13 (±8.73)	67.00 (±8.07)	t=-0.658; df=158; p=0.511
Nationality, number (%)				Χ^2^(2)=3.030; p=0.271*
Greek	110 (68.8%)	60 (75.0%)	50 (62.5%)	
Albanian	44 (27.5%)	18 (22.5%)	26 (32.5%)	
Cypriot	6 (3.8%)	2 (2.5%)	4 (5.0%)	
Marital status, number (%)				Χ^2^(2)=0.767; p=0.682
Married	110 (68.8%)	54 (67.5%)	56 (70.0%)	
Divorced	36 (22.5%)	20 (25.0%)	16 (20.0%)	
Single	14 (8.8%)	6 (7.5%)	8 (10.0%)	
Children, number (%)				Χ^2^(4)=4.716; p=0.331*
None	24 (15.0%)	12 (15.0%)	12 (15.0%)	
One	52 (32.5%)	32 (40.0%)	20 (25.0%)	
Two	58 (36.3%)	24 (30.0%)	34 (42.5%)	
Three	18 (11.3%)	8 (10.0%)	10 (12.5%)	
Four	8 (5.0%)	4 (5.0%)	4 (5.0%)	
Educational level, number (%)				Χ^2^(4)=3.011; p=0.556
Postgraduate studies	14 (8.8%)	8 (10.0%)	6 (7.5%)	
Higher education	38 (23.8%)	22 (27.5%)	16 (20.0%)	
Post-secondary education	36 (22.5%)	14 (17.5%)	22 (27.5%)	
Secondary education	48 (30.0%)	24 (30.0%)	24 (30.0%)	
Compulsory education	24 (15.0%)	12 (15.0%)	12 (15.0%)	
Employment status, number (%)				Χ^2^(2)=1.764; p=0.414
Unemployed	22 (13.8%)	12 (15.0%)	10 (12.5%)	
Retired	56 (35.0%)	24 (30.0%)	32 (40.0%)	
Employed	82 (51.2%)	44 (55.0%)	38 (47.5%)	

Clinical and postoperative characteristics

Clinical characteristics related to lung cancer diagnosis and surgical management were comparable between the two groups. No statistically significant differences were observed regarding type of surgical procedure (p=0.864), surgical approach (p=0.317), receipt of chemotherapy (p=0.260), radiotherapy (p=0.276), disease stage (p=0.959), postoperative complications (p=0.858), or readmission rates (p=0.798).

A statistically significant difference was observed in immunotherapy distribution between groups (p=0.014). Specifically, postoperative-only immunotherapy was more frequently reported in the control group (27, 33.8%) compared with the intervention group (14, 17.5%), whereas combined pre- and postoperative immunotherapy was more common in the intervention group (19, 23.8%) compared with the control group (10, 12.5%). Preoperative-only immunotherapy was observed exclusively in the intervention group (3, 3.8%), while the proportion of patients receiving no immunotherapy was similar between groups (43 (53.8%) versus 44 (55.0%)). These variables were therefore included in sensitivity analyses.

In addition, a statistically significant difference was observed in length of hospital stay, which was shorter in the intervention group compared with the control group (4.51±0.62 versus 5.71±1.18 days, p<0.001) (Table 2).

**Table 2:** Clinical and postoperative characteristics of the total sample, control group, and intervention group SD: standard deviation *Monte Carlo

Clinical and postoperative characteristics	Total sample	Control group	Intervention group	Comparison, p-value
Type of surgery, number (%)				Χ^2^(1)=0.029; p=0.864
Lobectomy	111 (69.4%)	55 (68.8%)	56 (70.0%)	
Segmentectomy or pneumonectomy	49 (30.6%)	25 (31.3%)	24 (30.0%)	
Surgical approach, number (%)				Χ^2^(1)=1.002; p=0.317
Thoracotomy	142 (88.8%)	69 (86.3%)	73 (91.3%)	
Thoracoscopic	18 (11.3%)	11 (13.8%)	7 (8.8%)	
Chemotherapy, number (%)				Χ^2^(3)=4.181; p=0.260*
Νο	73 (45.6%)	38 (47.5%)	35 (43.8%)	
Yes, preoperative only	4 (2.5%)	1 (1.3%)	3 (3.8%)	
Yes, postoperative only	53 (33.1%)	30 (37.5%)	23 (28.7%)	
Yes, preoperative and postoperative	30 (18.8%)	11 (13.8%)	19 (23.8%)	
Radiotherapy, number (%)				Χ^2^(1)=2.105; p=0.276*
No	152 (95.0%)	78 (97.5%)	74 (92.5%)	
Yes, postoperative	8 (5.0%)	2 (2.5%)	6 (7.5%)	
Immunotherapy, number (%)				Χ^2^(3)=9.927; p=0.014*
Νο	87 (54.4%)	43 (53.8%)	44 (55.0%)	
Yes, preoperative only	3 (1.9%)	0 (0.0%)	3 (3.8%)	
Yes, postoperative only	41 (25.6%)	27 (33.8%)	14 (17.5%)	
Yes, preoperative and postoperative	29 (18.1%)	10 (12.5%)	19 (23.8%)	
Disease stage, number (%)				Χ^2^(5)=1.037; p=0.959
ΙΑ	54 (33.8%)	27 (33.8%)	27 (33.8%)	
ΙΒ	24 (15.0%)	13 (16.3%)	11 (13.8%)	
ΙΙΑ	19 (11.9%)	10 (12.5%)	9 (11.3%)	
ΙΙΒ	28 (17.5%)	14 (17.5%)	14 (17.5%)	
ΙΙΙΑ	24 (15.0%)	12 (15.0%)	12 (15.0%)	
ΙΙΙΒ	11 (6.9%)	4 (5.0%)	7 (8.8%)	
Readmission, number (%)				Χ^2^(1)=0.066; p=0.798
No	143 (89.4%)	71 (88.8%)	72 (90.0%)	
Yes	17 (10.6%)	9 (11.3%)	8 (10.0%)	
Complications, number (%)				Χ^2^(1)=0.032; p=0.858
No	117 (73.1%)	59 (73.8%)	58 (72.5%)	
Yes	43 (26.9%)	21 (26.3%)	22 (27.5%)	
Length of hospital stay (days), mean (±SD)	5.11 (±1.12)	5.71 (±1.18)	4.51 (±0.62)	F(1, 158)=64.832; p<0.001

Pain-related outcomes

Primary Outcome: Current Pain Intensity

Linear mixed-effects analysis demonstrated a statistically significant main effect of group (F(1, 155.89)=109.42, p<0.001) and time (F(3, 200.53)=700.75, p<0.001), as well as a significant group × time interaction (F(3, 200.53)=27.92, p<0.001). These results indicate that pain trajectories over time differed significantly between the intervention and control groups.

At baseline, a statistically significant difference in current pain intensity was observed, with the control group reporting slightly higher scores than the intervention group (3.10±0.10 versus 2.79±0.10; mean difference: 0.31; 95% CI: 0.04-0.59; p=0.027).

**Figure 2:**
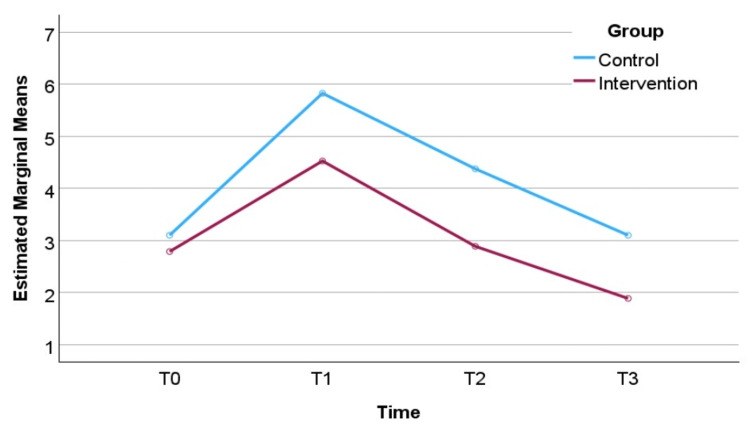
Estimated marginal means of current pain intensity across the study time points in the control and intervention groups

At postoperative follow-up assessments (T1, T2, and T3), the intervention group consistently demonstrated lower pain intensity scores compared with the control group. The greatest between-group difference was observed at T2 (4.38±0.09 versus 2.89±0.09; mean difference: 1.49; 95% CI: 1.24-1.73; p<0.001). The overall group × time interaction remained statistically significant after adjustment for baseline pain intensity (F(2, 198.23)=3.31, p=0.038), confirming the robustness of the findings (Table 3, Figure 2).

**Table 3:** Linear mixed model analysis of Pain Rating Scale scores across four time points in the control and intervention groups CG: control group, IG: intervention group, SE: standard error, CI: confidence interval, T0: baseline, T1: 1 month after surgery, T2: 6 months after surgery, T3: 12 months after surgery

Pain Rating Scale item	Statistic	T0	T1	T2	T3	Group effect	Time effect	Group × time effect
Current pain intensity	CC, mean (±SE)	3.10 (±0.10)	5.83 (±0.09)	4.38 (±0.09)	3.10 (±0.09)	F(1,155.89)=109.42; p<0.001	F(3,200.53)=700.75; p<0.001	F(3,200.53)=27.92; p<0.001
Current pain intensity	IG, mean (±SE)	2.79 (±0.10)	4.53 (±0.09)	2.89 (±0.09)	1.89 (±0.09)			
Current pain intensity	CC-IG mean difference (95% CI); p-value	0.33 (0.04, 0.59); p=0.027	1.30 (1.04, 1.56); p<0.001	1.49 (1.24, 1.73); p<0.001	1.21 (0.98, 1.49); p<0.001			
Average pain intensity during previous week	CC, mean (±SE)	3.04 (±0.10)	6.19 (±0.11)	4.38 (±0.09)	3.10 (±0.08)	F(1,155.90)=110.42; p<0.001	F(3,228.72)=637.80; p<0.001	F(3,228.72)=37.78; p<0.001
Average pain intensity during previous week	IG, mean (±SE)	2.89 (±0.10)	4.79 (±0.11)	2.89 (±0.09)	1.89 (±0.08)			
Average pain intensity during previous week	CC-IG mean difference (95% CI); p-value	0.15 (-0.12, 0.42); p=0.270	1.40 (1.11, 1.69); p<0.001	1.49 (1.24, 1.74); p<0.001	1.21 (0.99, 1.44); p<0.001			
Current pain distress	CC, mean (±SE)	3.49 (±0.10)	6.08 (±0.09)	4.66 (±0.09)	3.20 (±0.08)	F(1,157.30)=130.14; p<0.001	F(3,218.81)=574.79; p<0.001	F(3,218.81)=22.75; p<0.001
Current pain distress	IG, mean (±SE)	3.19 (±0.10)	4.71 (±0.09)	3.20 (±0.09)	2.06 (±0.08)			
Current pain distress	CC-IG mean difference (95% CI); p-value	0.30 (0.03, 0.57); p=0.032	1.36 (1.11, 1.62); p<0.001	1.46 (1.21, 1.71); p<0.001	1.14 (0.91, 1.37); p<0.001			
Average pain distress during previous week	CC, mean (±SE)	3.03 (±0.09)	6.18 (±0.11)	4.36 (±0.10)	3.09 (±0.08)	F(1,158.0)=112.65; p<0.001	F(3,158.0)=587.99; p<0.001	F(3,158.0)=34.15; p<0.001
Average pain distress during previous week	IG, mean (±SE)	2.88 (±0.09)	4.78 (±0.11)	2.88 (±0.10)	1.88 (±0.08)			
Average pain distress during previous week	CC-IG mean difference (95% CI); p-value	0.15 (-0.11, 0.41); p=0.136	1.40 (1.10, 1.69); p<0.001	1.48 (1.23, 1.73); p<0.001	1.21 (0.98, 1.44); p<0.001			
Interference of pain with daily activities	CC, mean (±SE)	3.45 (±0.06)	5.05 (±0.08)	4.10 (±0.06)	3.41 (±0.07)	F(1,166.13)=56.02; p<0.001	F(3,222.33)=263.44; p<0.001	F(3,222.33)=26.47; p<0.001
Interference of pain with daily activities	IG, mean (±SE)	3.38 (±0.06)	4.41 (±0.08)	3.30 (±0.06)	2.79 (±0.07)			
Interference of pain with daily activities	CC-IG mean difference (95% CI); p-value	0.08 (-0.10, 0.25); p=0.398	0.64 (0.41, 0.87); p<0.001	0.80 (0.64, 0.96); p<0.001	0.63 (0.44, 0.81); p<0.001			

Secondary outcomes

Average Pain Intensity (Previous Week)

Statistically significant main effects of group (F(1, 155.90)=110.42, p<0.001) and time (F(3, 228.72)=637.80, p<0.001), as well as a significant group × time interaction (F(3, 228.72)=37.78, p<0.001), were observed.

**Figure 3:**
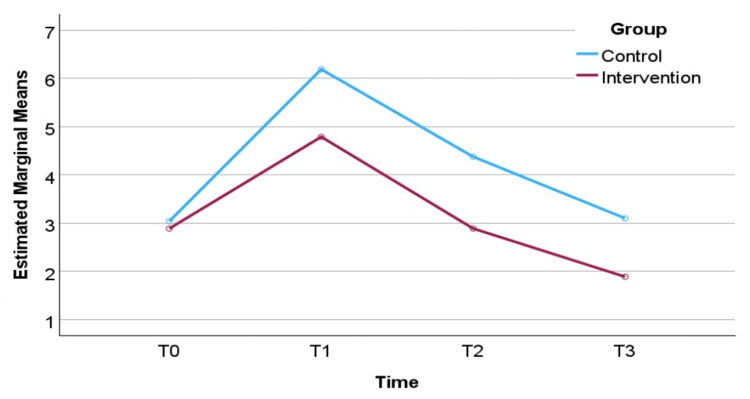
Estimated marginal means of average pain intensity during the previous week across the study time points in the control and intervention groups

No significant baseline difference was identified between groups (3.04±0.10 versus 2.89±0.10; p=0.270). From T1 onward, the intervention group showed consistently lower pain intensity compared with the control group. The largest difference was observed at T2 (4.38±0.09 versus 2.89±0.09; p<0.001), followed by sustained differences at T3 (Table 3, Figure 3).

Current Pain Distress

Analysis revealed significant effects of group (F(1, 157.30)=130.14, p<0.001), time (F(3, 218.81)=574.79, p<0.001), and a significant group × time interaction (F(3, 218.81)=22.75, p<0.001).

At baseline, the control group reported slightly higher pain distress than the intervention group (3.49±0.10 versus 3.19±0.10; p=0.032). During follow-up, the intervention group consistently demonstrated lower pain distress scores at all time points, with the largest difference observed at T2 (4.66±0.09 versus 3.20±0.09; p<0.001).

**Figure 4:**
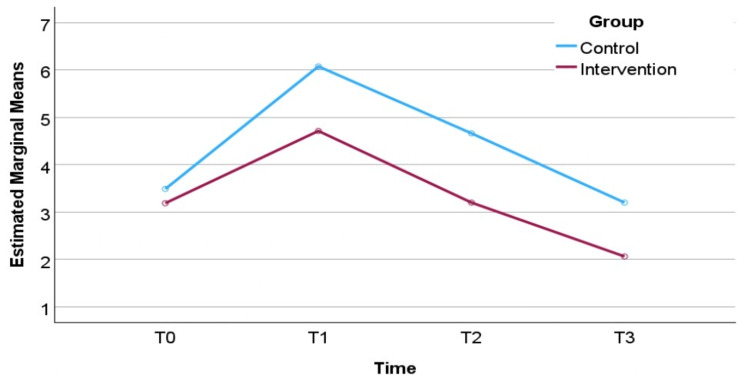
Estimated marginal means of current pain distress across the study time points in the control and intervention groups

After adjustment for baseline pain distress, the group × time interaction remained statistically significant (F(2, 211.02)=3.37, p=0.036), indicating persistence of the intervention effect (Table 3, Figure 4).

Average Pain Distress (Previous Week)

Statistically significant main effects of group and time (both p<0.001) and a significant group × time interaction (F(3, 158.0)=34.15; p<0.001) were observed.

**Figure 5:**
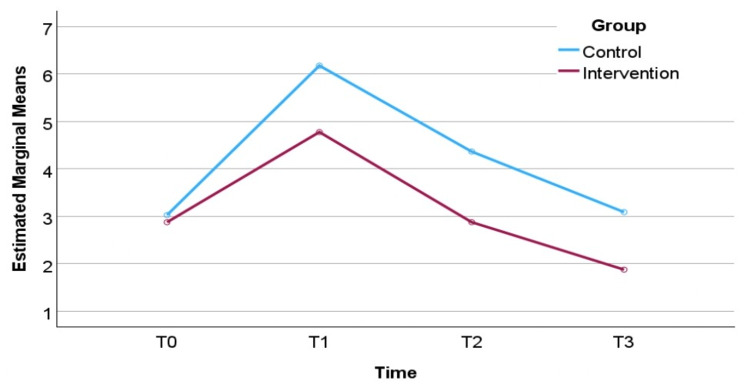
Estimated marginal means of average pain distress during the previous week across the study time points in the control and intervention groups

No baseline differences were detected between groups (p=0.136). From T1 onward, the intervention group exhibited consistently lower pain distress scores compared with controls, with the greatest reduction observed at T2 and sustained effects through T3 (Table 3, Figure 5).

Pain-Related Interference With Daily Activities

Significant effects of group (F(1, 166.13)=56.02, p<0.001), time (F(3, 222.33)=263.44, p<0.001), and a group × time interaction (F(3, 222.33)=26.47, p<0.001) were identified.

**Figure 6:**
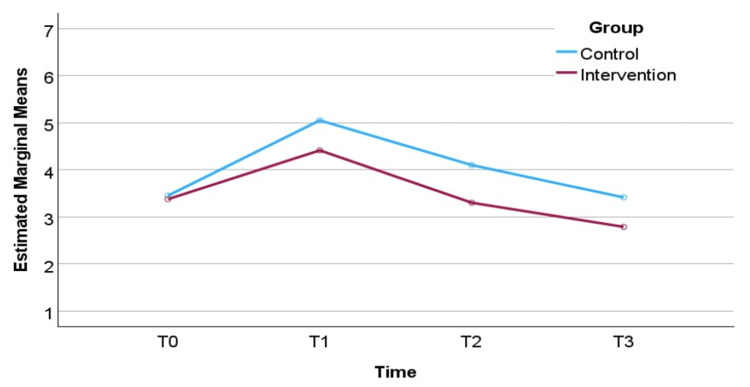
Estimated marginal means of pain-related interference with daily activities across the study time points in the control and intervention groups

Baseline scores did not differ significantly between groups (3.45±0.06 versus 3.38±0.06; p=0.398). At follow-up, the intervention group consistently demonstrated lower interference with daily activities across all time points, with the largest effect observed at T2 (4.10±0.06 versus 3.30±0.06; p<0.001) (Table 3, Figure 6).

Sensitivity Analyses

Sensitivity analyses adjusted for length of hospital stay and immunotherapy status confirmed the robustness of all main findings. The group × time interactions for all pain-related outcomes remained statistically significant after adjustment, indicating that the observed effects were not materially influenced by baseline imbalances in these variables.

Summary of Main Findings

Overall, patients receiving the structured perioperative educational intervention demonstrated significantly improved postoperative pain trajectories compared with the control group. This included lower pain intensity, reduced pain distress, and decreased interference of pain with daily functioning over a 12-month follow-up period. The consistent and significant group × time interactions across outcomes indicate distinct recovery patterns between groups.

## Discussion

This prospective non-randomized interventional study demonstrated that a structured perioperative educational intervention was associated with significantly improved postoperative pain-related outcomes over a 12-month follow-up period in patients undergoing lung cancer surgery. Compared with patients receiving usual care alone, participants in the intervention group consistently reported lower pain intensity, lower pain distress, and reduced pain-related interference with daily activities across postoperative follow-up assessments.

Current pain intensity demonstrated a significantly different postoperative trajectory between groups, with consistently lower scores in the intervention group across follow-up assessments. This finding remained robust after adjustment for baseline differences, supporting the stability of the observed association. These results are consistent with previous evidence suggesting that perioperative education may contribute to improved postoperative pain outcomes in thoracic surgery populations [5,24]. A plausible explanation is that structured education enhances patients’ understanding of expected postoperative pain, facilitates earlier recognition and reporting of symptoms, and promotes timely use of analgesic strategies, thereby improving pain control [25].

Average pain intensity during the previous week, a more stable indicator of persistent postoperative pain experience, was also significantly lower in the intervention group across follow-up time points. This finding is particularly relevant given the clinical importance of persistent post-surgical pain following lung cancer surgery [26,27]. The sustained differences observed up to 12 months suggest that the intervention may have influenced longer-term pain processing and adaptation, potentially through improved self-management behaviors and adherence to rehabilitation strategies such as mobilization and respiratory exercises.

Pain distress was significantly reduced in the intervention group, indicating that the educational intervention influenced not only the sensory dimension of pain but also its affective component. This finding may reflect reduced uncertainty regarding the postoperative course and improved psychological preparedness, which are known to mitigate pain catastrophizing and anxiety-related amplification of pain perception [28]. These results align with prior studies that highlight the role of structured education in reducing emotional distress and improving coping in surgical oncology populations [5].

Pain-related interference with daily activities was also significantly lower in the intervention group, suggesting improved functional recovery. The incorporation of behavioral guidance, including early mobilization, breathing exercises, and structured self-care strategies, may have reduced fear of movement and facilitated earlier return to functional independence [26,29]. This finding is consistent with evidence supporting multimodal perioperative rehabilitation strategies that integrate education and behavioral support to enhance recovery outcomes [30,31].

The findings of the present study support the growing recognition that perioperative care in thoracic oncology should extend beyond surgical management alone and incorporate supportive interventions targeting behavioral and psychological recovery. Enhanced Recovery After Surgery (ERAS) programs have consistently been associated with improved perioperative outcomes, including shorter hospital stay and reduced postoperative complications in thoracic surgery. More recently, Comfort-Enhanced Recovery After Surgery (ComERAS) approaches have expanded traditional ERAS principles by placing greater emphasis on patient comfort and patient-reported outcomes throughout the perioperative period. In addition to multimodal perioperative management, ComERAS pathways incorporate coordinated multidisciplinary care, structured patient support, and standardized recovery targets related to analgesia, mobilization, respiratory rehabilitation, and postoperative care. Although the present study did not evaluate a formal ERAS or ComERAS program, the observed benefits of structured perioperative education are consistent with the broader goal of promoting patient-centered recovery and improving postoperative symptom management after lung cancer surgery [32].

Importantly, baseline differences in immunotherapy exposure and length of hospital stay were observed between groups, reflecting the non-randomized design and potential for residual confounding. However, sensitivity analyses adjusting for these variables did not materially alter the observed associations, supporting the robustness of the findings. Notably, the shorter hospital stay observed in the intervention group may reflect improved postoperative engagement and earlier mobilization, although causal inference is limited due to the non-randomized study design.

Overall, the findings support the integration of structured perioperative educational interventions into standard care pathways for patients undergoing lung cancer surgery. Such interventions represent a low-cost and feasible supportive strategy that may enhance postoperative recovery by targeting cognitive, behavioral, and emotional determinants of pain experience. Further randomized, multicenter studies are warranted to confirm these findings and to explore long-term outcomes, including chronic post-surgical pain, analgesic consumption, functional recovery, and quality of life.

Importantly, although the observed associations remained consistent across multiple pain-related outcomes and persisted after adjustment for baseline imbalances and clinically relevant covariates, the non-randomized design precludes definitive causal inference. Consequently, the findings should be interpreted as evidence of a potentially beneficial association between structured perioperative education and postoperative pain outcomes, pending confirmation in adequately powered randomized controlled trials.

Limitations

This study has several limitations that should be acknowledged. First, the single-center non-randomized design limits the generalizability of the findings and introduces the possibility of residual confounding despite the use of adjusted analyses and sensitivity analyses. Second, blinding of participants was not feasible due to the nature of the educational intervention, which may have introduced response bias in self-reported outcomes. Third, pain outcomes were based on patient-reported measures and may therefore be influenced by subjective perception and reporting tendencies. In addition, no formal a priori sample size calculation was performed, which may limit the statistical precision of the findings.

Although the present analysis did not include a comprehensive assessment of quality of life, the pain assessment tool used in this study captured several pain-related dimensions that are closely linked to daily functioning and perceived recovery, including pain intensity, pain-related distress, and interference with daily activities. Consequently, the findings provide insight into important pain-related aspects of patient-centered recovery, while future studies should incorporate validated quality-of-life instruments to assess the broader impact of educational interventions. Therefore, although the results suggest a beneficial effect of the educational intervention on postoperative pain outcomes, further adequately powered, multicenter randomized controlled trials are required to confirm these findings and evaluate additional patient-centered outcomes.

Finally, although statistically significant associations were observed across all pain-related outcomes and remained robust in multiple sensitivity analyses, the non-randomized design precludes definitive causal inference. Therefore, the findings should be interpreted as evidence of an association between structured perioperative education and improved postoperative pain-related outcomes rather than proof of causality.

## Conclusions

This study found that a structured educational intervention was associated with improved postoperative pain-related outcomes among patients undergoing surgery for lung cancer. Compared with usual care alone, the intervention was associated with lower current pain intensity, lower average pain intensity during the previous week, reduced pain distress, and less pain-related interference with daily activities over time. These results suggest that structured perioperative education, reinforced during the early postoperative period and supported by caregiver involvement, may represent a useful, feasible, and low-risk strategy to improve pain management after lung cancer surgery. Further multicenter randomized studies are warranted to confirm these findings and evaluate long-term effects on chronic post-surgical pain, rehabilitation outcomes, and quality of life.

## Figures and Tables

**Figure 7:**
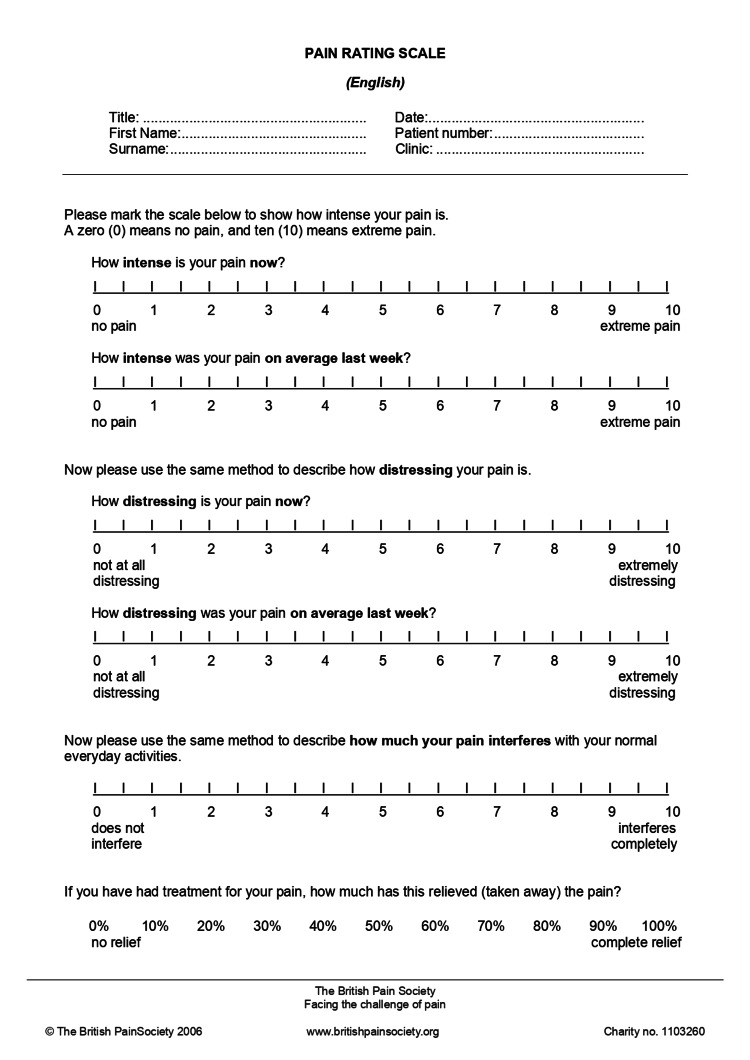
Pain scale questionnaire administered to patients

## Appendices

Figure 7 presents the pain assessment scale used in this study.  
